# Logic in the time of coronavirus

**DOI:** 10.1099/jmm.0.001191

**Published:** 2020-04-22

**Authors:** Timothy J. J. Inglis

**Affiliations:** ^1^​ Division of Pathology and Laboratory Medicine, School of Medicine, University of Western Australia, Western Australia, Australia; ^2^​ Department of Microbiology, PathWest Laboratory Medicine WA, Nedlands, WA 6009, Australia

**Keywords:** COVID-19, SARS-CoV-2, coronavirus, principles of aetiology, countermeasures

## Abstract

Much has happened here since the local news media trumpeted the first Australian COVID-19 fatality, and stirred up a medieval fear of contagion. We now need to take a step back to examine the logic underlying the use of our limited COVID-19 countermeasures. Emerging infectious diseases by their nature, pose new challenges to the diagnostic-treatment-control nexus, and push our concepts of causality beyond the limits of the conventional Koch-Henle approach to aetiology. We need to use contemporary methods of assessing causality to ensure that clinical, laboratory and public health measures draw on a rational, evidence-based approach to argumentation. The purpose of any aetiological hypothesis is to derive actionable insights into this latest emerging infectious disease. This review is an introduction to a conversation with medical microbiologists, which will be supported by a moderated blog.

Much has happened here since the local news media trumpeted the first Australian COVID-19 fatality, stirred up a medieval fear of contagion and squeezed the pips out of scientific enlightenment. Yet the calm pragmatism of professional colleagues, and their dogged determination to sustain a diagnostic service in difficult circumstances, has been a dignified fixed point in these troubled times.

We need now to take a step back and examine the logic underlying the use of our limited suite of COVID-19 countermeasures. In particular, to avoid media-led confusion, we should respect the boundaries between illness, objectively verified disease and laboratory-confirmed infection. We are now relying on nucleic acid amplification tests to confirm recent infection with exquisite sensitivity, while hoping that primer and probe-binding viral sequences do not mutate beyond recognition. This level of technical sensitivity may suit the demands of public health agencies grappling with disease control, but diagnostic specificity across the varied range of COVID-19 presentations, and different stages in their clinical evolution, is less clear. As a result, we are in the invidious position of having to chase a moving viral target in a rapidly escalating pandemic without the full set of test performance, clinical and epidemiological data that we normally rely on. And with our eyes on a single diagnostic target, there is a fair chance we may miss other treatable infectious diseases in plain sight. Hence, in addition to the nucleic acid detection assays, there is also great interest in the development and assessment of serological assays for SARS-CoV-2 to assess previous infection and possible immunity and for seroincidence surveys. Those involved with existing microbiological testing will appreciate that the characteristics of analytical and clinical sensitivity and specificity, positive and negative predictive values and correlates of protection will be challenging to determine.

Emerging infectious diseases, by their very nature, pose new challenges to the diagnostic–treatment–control nexus, and push our concepts of causality to the limit. The conventional Koch–Henle approach to aetiology offers little help here. Instead, we need to use more contemporary methods of assessing causality to make sense of its inherent complexity [1], so that clinical, laboratory and public health measures can draw on a rational, evidence-based approach to argumentation. There are enough data at this stage in the COVID-19 epidemic to start populating a cohesive cause and effect narrative: congruence (convergence of molecular biology, clinical features and epidemiology), consistency (reproducible features of this nexus, and its temporal–spatial clustering), cumulative dissonance (upscaling through multiple levels of biological organization) and curtailment (diagnosis, containment, control and preventive interventions). A formal review of the argument for causality goes much further than attribution of a pathogenic role for a named microbe. The original purpose of the priobe hypothesis was, and remains, the derivation of actionable insights into emerging infectious diseases. As the COVID-19 pandemic is the latest substantial emerging infectious disease challenge, the priobe hypothesis provides a prism through which to view this novel disease and its causal agent and to determine priorities for evidence-based action. This short review serves as an introduction to a conversation between medical microbiologists and their colleagues, to be supported by a moderated blog that will be updated with new insights as they appear in the scientific literature.

We are seeing measures against COVID-19 that hark back to the 1918–19 influenza pandemic and the great plague outbreaks. When armed troops are deployed on our streets, public health staff in protective suits and respirators spray the streets with disinfectant, and public meetings of more than two people are cancelled, the normal rhythms of life have been disturbed. As clinician–scientists, it is our responsibility to tackle the challenge with the skills and insights we know best, to obtain an accurate measure of the threat and shape an effective, data-driven response and to educate our colleagues accordingly. Before we resort to superstition-driven 14th century concepts of disease control, let us take a hard look at the data, mine them with the best machine learning tools at hand, and make pragmatic decisions about how COVID-19’s remorseless progress can be disrupted within existing resources.

## Congruence

### Molecular biology

The pandemic of COVID-19 has been attributed to a novel SARS-like coronavirus that differs from the original by 380 amino acid substitutions [2]. This novel coronavirus has been named SARS-CoV-2 [3].

### Clinical features

The clinical syndrome associated with SARS-CoV-2 comprises a group of clinical features varying in clinical severity from an upper respiratory tract infection to lung infection requiring supplementary oxygen and, in severe cases, intensive care [4]. In the Singapore study, SARS-CoV-2 remained detectable for 7 or more days, and the anti-retroviral agent lopinavir/ritonavir was used for treatment without any deaths.

Computed tomography has proved to be useful as a diagnostic adjunct in patients with lower respiratory tract infection [5].

### Epidemiology

Given the wealth of epidemiological data emerging from multiple sources, a group at the Harvard Center for Communicable Disease Dynamics identified key unanswered questions [6]. Among the most important questions are the incubation period and the reproduction number. Estimates of the incubation period vary with a median around 5 days, but a period of 14 days or more has been reported [7–9]. Another key measure is the serial interval between consecutive cases, estimated as just under 5 days [10]. The other measure of transmission closely watched is the reproduction number; the number of secondary cases arising after transmission from an index case. In the early stages of the pandemic this was estimated at 2.9 [11].

## Consistency

A report on the first case cluster of novel coronavirus infection implicated a seafood market in Wuhan, PR China [12]. Subsequent spread out of Wuhan province was then associated with propagation of epidemic SARS-CoV-2 infection [13]. In other parts of China, a similar disease spectrum emerged, with a notable reduction in the estimated mortality rate [14]. Once outside China, COVID-19 spread was rapid, leading to new national epidemics in distant locations such as the World Health Organization (WHO) European region [15]. A consistent clinical picture emerged in which more severe infections affect older patients with cough, expectoration, chest pain, dyspnoea and a higher computed tomography (CT) score [16]. Indeed, CT appearance appears to correlate with SARS-CoV-2 PCR assay results [17]. We can expect further reports of the common features of COVID-19 case clusters, national outbreaks and their corresponding laboratory investigations in weeks to come.

## Cumulative dissonance

The insights that connect the different levels of biological organization from the molecular level to organ systems, people and their communities are scarce as yet. They do not permit detailed understanding of how and why an initial encounter with SARS-CoV-2 produces its typical clinico-pathological effects. The processes that underlie escalation through multiple layers of increasing complexity remain opaque at this point, but glimpses of the molecular mechanisms of cell penetration and damage are beginning to emerge. There is a structural basis for cellular recognition of SARS-CoV-2 [18], a potentially blockable cell entry pathway [19], and a mouse model of coronavirus infection that may be a small step towards establishing causality [20]. From what is already known about the severity stratification of COVID-19, it is possible to infer a clinico-pathological progression to more extensive cell, tissue and organ damage in a diminishing proportion of patients, but we are a long way short of a mechanistic construct of the process.

## Curtailment

The logical conclusion of any analysis of an emerging infectious disease is actionable insights; a set of diagnostic, treatment, control, containment and preventive measures. At present, the available measures are principally containment and control by physical distancing and improved hygiene. Their efficacy has been the subject of intense debate in view of the disruption they caused in the main centres of infection, but evidence has been presented to justify the control measures taken in China [21] and a case has been made for wider application of containment and control measures in other countries using mathematical modelling [22]. Meanwhile, patients at the severe and critical end of the COVID-19 spectrum are being treated on a compassionate, experimental basis with the antiretrovial drugs lopinavir/ritonavir [4, 23]. An expert consensus from the epicentre of the original COVID-19 outbreak has recommended the use of chloroquine for treatment of mild, moderate and severe pneumonia during the current outbreak [24]. As these findings have been rebutted by other groups, there is obviously a need for careful assessment [25]. Clearly, it will take too long to develop novel pharmaceutical products targeted at SARS-CoV-2, but repurposing existing products that are widely available is eminently feasible if clinical benefit can be demonstrated in suitable trials. Finally, work has commenced on vaccine development [26]. Whilst welcome, these initiatives will likely take too long to be useful in the short term, but may have a use in the prevention of future epidemics, or in targeted prevention among highly vulnerable populations.

The weakest links in the chain of causation appear to concern progression in affected patients from the initial coronavirus encounter, through an inferred viraemia, to escalating cell, tissue and organ damage. A better understanding of the mechanisms of progression or cumulative dissonance should lead us to additional interventional insights, thus aiding the assembly of a cohesive COVID-19 defence strategy. This approach, as previously applied during local emerging infection events [1], may be what we need to identify COVID-19’s centre of gravity and critical vulnerabilities. In this rapidly developing field, we shall be discussing further aspects of COVID-19 on the Microbiology Society blog.

### For discussion on the *Journal of Medical Microbiology* blog

#### Congruence

1. Best use of limited RT-PCR assays as the pandemic evolves.
2. Clinical pathology and diagnostic imaging surrogates for PCR assay-based diagnosis.
3. Molecular epidemiology of SARS-CoV-2.

#### Consistency

1. What, why and how co-morbidities affect COVID-19 outcomes.

#### Cumulative dissonance

1. Tissue pathology features of COVID-19.
2. Mechanisms of escalating clinico-pathological consequences of SARS-CoV-2 infection.
3. SARS-CoV-2 transmissibility/virulence trade-off.

#### Curtailment

1. Triggers for a phased approach to containment and control of COVID-19.
2. SARS-CoV-2 persistence on inanimate surfaces and decontamination methods.
